# Immobilization of a TiO_2_–PEDOT:PSS hybrid heterojunction photocatalyst for degradation of organic effluents

**DOI:** 10.1039/d2ra06729c

**Published:** 2023-01-19

**Authors:** Durga Sankar Vavilapalli, Johanna Rosen, Shubra Singh

**Affiliations:** a Materials Design, Department of Physics, Chemistry and Biology (IFM), Linköping University SE-581 83 Linköping Sweden durga.sankar.vavilapalli@liu.se; b Crystal Growth Centre, Alagappa College of Technology (AcTech) Campus, Anna University Chennai-600025 India; c Centre for Energy Storage Technologies, Anna University Chennai-600025 India

## Abstract

Heterojunction photocatalysts have recently emerged for use in degradation of organic pollutants, typically being suspended in effluent solution to degrade it. Post degradation, the catalyst must be removed from the treated solution, which consumes both energy and time. Moreover, the separation of nano catalysts from the treated solution is challenging. In the present work, we explore fabrication of immobilized TiO_2_–PEDOT:PSS hybrid heterojunction catalysts with the support of a PVA (polyvinyl alcohol) matrix. These photocatalytic films do not require any steps to separate the powdered catalyst from the treated water. While the PVA-based films are unstable in water, their stability could be significantly enhanced by heat treatment, enabling efficient removal of organic effluents like methylene blue (MB) and bisphenol-A (BPA) from the aqueous solution under simulated sunlight irradiation. Over 20 cycles, the heterojunction photocatalyst maintained high photocatalytic activity and showed excellent stability. Hence, an immobilization of the TiO_2_–PEDOT:PSS hybrid heterojunction is suggested to be beneficial from the viewpoint of reproducible and recyclable materials for simple and efficient wastewater treatment.

## Abstract 2

A plausible mechanism of photodegradation of methylene blue using a PVA/TiO_2_–PEDOT:PSS heterojunction catalyst film.

## Graphical abstract

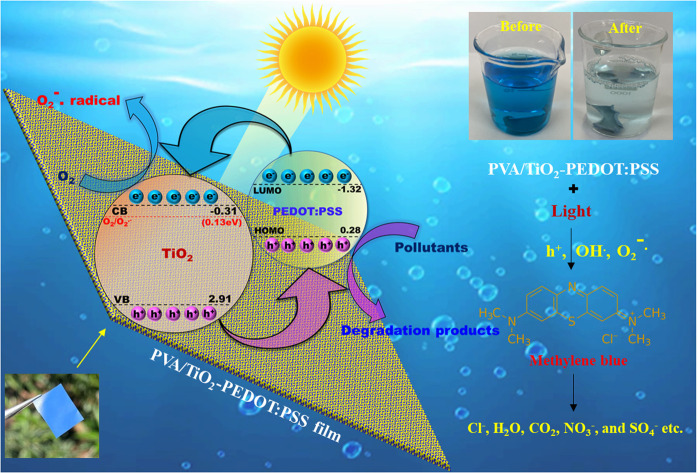

## 1. Introduction

Titanium dioxide (TiO_2_), being one of the promising semiconductor photocatalysts, is widely used to degrade many organic pollutants and is highly rated due to its catalytic reactivity, physical and chemical stability, nontoxicity and low-cost.^1–5^ In conventional wastewater treatment processes, TiO_2_ nanoparticles (NPs) are directly dispersed in the effluent solution, providing a high catalyst surface area which reacts with the pollutant.^6^ However, this mechanism possesses some practical technical challenges. Separation of TiO_2_ nanoparticles from the solution post-treatment remains a major obstacle, because of the required time, energy, and high cost, which limits the conventional photocatalytic degradation process for practical usage.^6,7^ For industrial applications, the photocatalysts are expected to be recyclable, for use in multiple cycles and with no loss of TiO_2_ nanoparticles during the long-term process of recycling.^8^ Immobilizing TiO_2_ nanoparticles on a variety of substrates is a promising method to overcome the current limitations.^9,10^ Furthermore, immobilization of the catalyst with a polymer matrix support could be advantageous over the other methods, since hydroxyl groups or carboxyl groups on the polymers may form chemical bonding with the hydroxyl groups on the surface of the TiO_2_ nanoparticles.^11^ This is supported by a few previous reports on immobilization of TiO_2_ NPs using polymer matrices.^11–16^ Polyvinyl alcohol (PVA) is a hydrophilic polymer with good flexibility and low cost and could also be potential candidate to immobilize TiO_2_.^17,18^ The swelling of PVA after being in contact with an aqueous liquid may facilitate an increase in the interface area between catalyst and effluent^19–21^ which would be advantageous compared to conventional substrate (*e.g.*, glass, metal *etc.*) supported catalyst immobilization.^6,22–26^

The wide bandgap of TiO_2_ (∼3.2 eV) poses another limitation to its absorption capability of incident light. TiO_2_ can absorb only UV-light, which is 3–5% of the sunlight coming to earth.^27–31^ Forming a heterojunction with another lower bandgap semiconductor could be one of the alternatives to reduce the effective bandgap, so that it can absorb more sunlight.^32^ A heterojunction between two semiconductors with matching electronic band structures can significantly reduce the effective bandgap and enhance the separation of photogenerated electrons (e^−^) and holes (h^+^), thereby considerably improving the photocatalytic performance.^32,33^ The organic conjugated polymer PEDOT:PSS (poly(3,4-ethylenedioxythiophene:polystyrene sulfonate)) which has been used as hole transport layer or as p-type material in organic electronics,^34,35^ may serve as a potential candidate for heterojunction semiconductors. PEDOT:PSS provides a good hole transport layer, with a low band gap (∼1.6 eV).^36^ It is thus expected that PEDOT:PSS (p-type) and TiO_2_ (n-type) can form a hybrid heterojunction due to their electronic band structure compatibility.^37^ The photoexcited electrons and holes in TiO_2_–PEDOT:PSS hybrid heterojunction can be localized at one semiconductor having more cathodic conduction band (CB) level and the other semiconductor possessing more anodic valence band (VB) level respectively. It provides Z-scheme charge transfer through heterostructures with superior reducing and oxidizing ability for enhancing the photocatalytic degradation performance.^38^

In this work, we demonstrate the fabrication of a TiO_2_–PEDOT:PSS hybrid heterojunction photocatalyst, to enhance the optical and electronic properties of an immobilized catalyst with the support of a PVA matrix. The photocatalytic performance of as fabricated TiO_2_–PEDOT:PSS and bare TiO_2_ hybrid films was investigated by degrading organic effluents methylene blue (MB) and bisphenol-A (BPA) under simulated sunlight.

## 2. Experimental

### 2.1 Preparation of TiO_2_–PEDOT:PSS hybrid heterojunction films

**Fig. 1:**
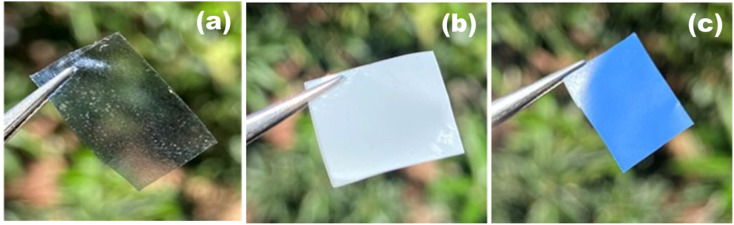
Photographs of (a) PVA/PEDOT:PSS (P), (b) PVA/TiO_2_ (T) and (c) PVA/TiO_2_–PEDOT:PSS (PT) films.

TiO_2_ (P25) nanoparticles (100 mg) were initially dispersed in 10 ml of DI water and ultrasonicated for 2 h 1 ml of PEDOT:PSS (Sigma Aldrich—1.3%) was mixed with the TiO_2_/H_2_O suspension after first being stirred magnetically at room temperature for 3 h 1 g of PVA was subsequently added into the TiO_2_–PEDOT:PSS solution under continuous stirring at 90 °C for 1 h, followed by stirring at 60 °C for 2 h. The solution was left in the beaker to avoid air bubbles and bring it down to room temperature. The PVA/TiO_2_–PEDOT:PSS solution was casted on Petri dishes and left overnight. For comparative purposes, bare PVA/TiO_2_ and PVA/PEDOT:PSS films were also prepared following the same procedure. The as-prepared dried films were heated at an optimized temperature of 140 °C for 2 h in vacuum. The experimental conditions like TiO_2_ to PVA weight ratio and thermal treatment temperature were systematically varied. It was found that 1 : 10 and 140 °C (2 h) were optimal conditions for fabricating stable and efficient hybrid heterojunction films. Furthermore, after identifying these optimal conditions, PEDOT:PSS Sigma Aldrich (1.3%) content was varied from 0.5 ml to 3 ml in 100 mg/10 ml TiO_2_–PVA suspension. We found that 1 ml of PEDOT:PSS in 10 ml of TiO_2_–PVA suspension was optimal for the formation of stable TiO_2_–PEDOT:PSS films, and showed better photocatalytic performance over other compositions. PVA/PEDOT:PSS, PVA/TiO_2_ and PVA/TiO_2_–PEDOT:PSS films were designated as P, T and PT respectively. Pictures of the as-prepared films are shown in Fig. 1. T-film appears in white colour and P-film appears in dark blue colour. Post mixing different weight percentages of TiO_2_ and PEDOT:PSS in order to form PT heterostructure, the colour of the heterostructure changes to whitish blue. Here the colours T and PT films are from precursor solution.

### 2.2 Characterization

The surface morphology and cross-sectional thickness of the as prepared films were examined using scanning electron microscopy (SEM, Jeol, 20 kV). The structural properties of the P, T and PT films were analyzed by X-ray diffraction (XRD, Bruker D2 X-ray Diffractometer, Cu Kα source, 2*θ* = 10–80°). The chemical structure and interaction between the PVA, TiO_2_ and PEDOT:PSS components were confirmed using Fourier Transfer Infra-Red Spectroscopy (FTIR, JASCO-5300 FT-IR spectrometer, within the range 200 to 4000 cm^−1^) and Raman spectroscopy (LabRAM HR Evolution, HORIBA, excitation wavelengths of 532 nm). During the photocatalytic degradation of the organic effluents, the effluent concentration was analyzed using UV-visible spectroscopy (Jasco V-730).

### 2.3 Photocatalytic degradation experiments

The photocatalytic degradation of methylene blue (MB) and bisphenol-A (BPA) was studied using PVA/TiO_2_ and PVA/TiO_2_–PEDOT:PSS hybrid films under simulated sunlight at standard test conditions (STC) (300 W, Xe-lamp source with 1000 W m^−2^ (1 sun) irradiation, AM1.5). Initially, 10 ppm of MB dye and BPA solutions were prepared, and 1.5 cm × 1.5 cm films were dipped into 20 ml of effluent solution and kept in the dark for 1 h to achieve an adsorption–desorption equilibrium. At every 1 h time interval, 10 ml of effluent solution was collected. The concentration of MB and BPA was then detected by measuring the maximum absorbance at 664 nm and 275 nm respectively, using UV-visible absorption spectroscopy (Jasco, V-730). The hybrid film after the degradation was washed and dried before another photodegradation test. The reusability of the photocatalytic film was tested for 20 cycles.

## 3. Results and discussion

**Fig. 2:**
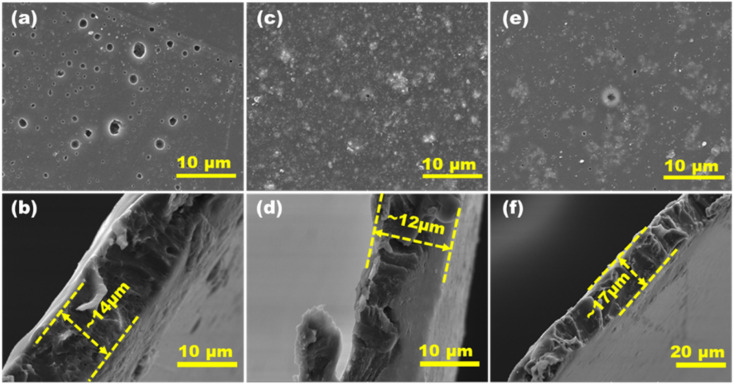
Surface morphology and corresponding cross-sectional images for the films P (a and b), T (c and d) and PT (e and f).

**Fig. 3:**
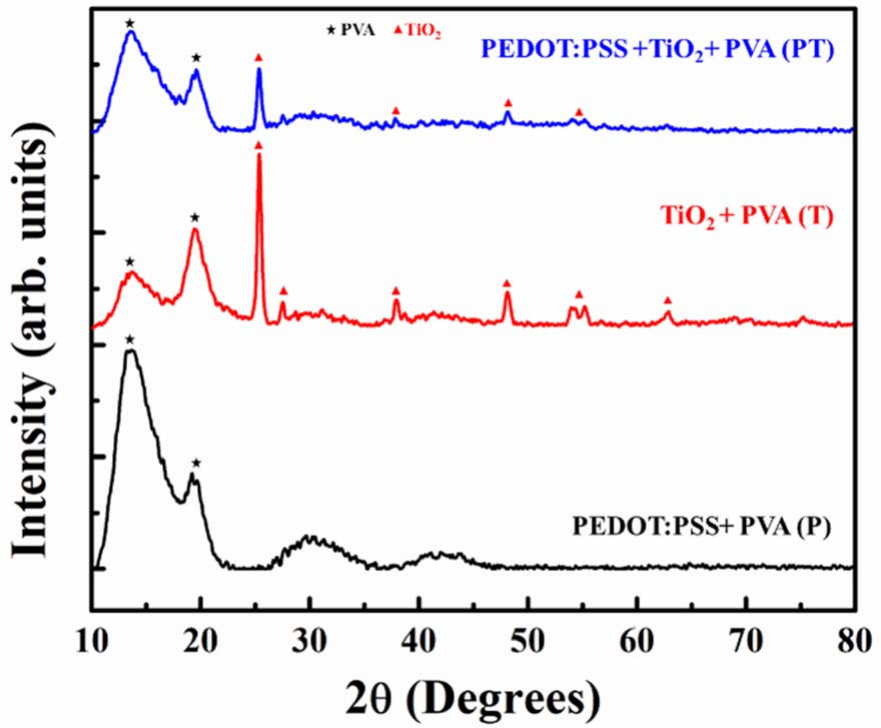
XRD data for the P, T and PT films.

Fig. 2(a–f) shows the surface morphology and corresponding cross-sectional images of the P, T and PT films. The surface morphology of the P film shows a uniform dispersion of PEDOT:PSS and the corresponding thickness of film is ∼14 μm. An aggregation of TiO_2_ NPs is observed in T and PT films with a cross sectional thickness of ∼12 μm and ∼17 μm respectively. All films have a uniform microporous surface structure due to heat treatment. The Energy Dispersive Spectroscopy (EDS) mapping shows (Fig. S1) a uniform distribution of Ti, O and S elements in the PT film. The XRD patterns of the P, T and PT films are shown in Fig. 3. In the XRD pattern of the P film, diffraction peaks corresponding to PEDOT:PSS are overlapped by the stronger peaks of PVA, while the XRD data for the T and PT films only show the characteristic peaks of PVA and TiO_2_. For complementary analysis, the existence of PEDOT:PSS and crosslinking of TiO_2_ and PVA in the PT film was confirmed by FTIR and Raman spectroscopy.

**Fig. 4:**
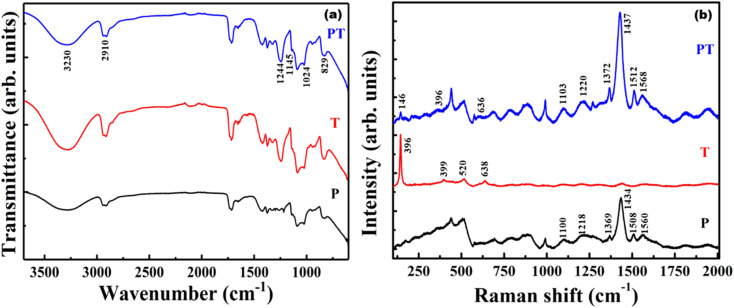
(a) FTIR spectra, (b) Raman spectra of P, T and PT films.

FTIR studies (Fig. 4(a)) were performed on P, T and PT films, to confirm the crosslinking of TiO_2_ NPs and PEDOT:PSS in a PVA matrix in order to immobilize the TiO_2_–PEDOT:PSS hybrid heterojunction catalyst. The stronger peaks corresponding to PVA overlap the corresponding peaks of PDOT:PSS. Hence, the signature peaks of PEDOT:PSS could not be identified in the FTIR spectra from the P and PT films. However, significant crosslinking between PVA and TiO_2_ was observed. The peak appearing at 1244 cm^−1^ in T and PT is from the vibration of Ti–O–C bonds,^11,39^ whereas corresponding peak in sample P is absent. Other smaller peaks at 1024 cm^−1^ and 829 cm^−1^ are attributed to O–C–C and C–C, respectively.^40^ It is expected that the PVA chains absorb heat and rearrange to form crystalline regions, leading to the appearance of a small peak at 1145 cm^−1^, corresponding to the C–C stretching vibration of the PVA crystals.^41^ These characteristic peaks indicate the crystallinity of the heat-treated P, T and PT samples. It also demonstrates the formation of covalent Ti–O–C bonds between PVA and TiO_2_ nanoparticles in the T and PT samples, which leads to effective immobilization of TiO_2_ nanoparticles in a highly stable PVA matrix. Under heat treatment, active OH groups located on the surface of nano-sized TiO_2_ and OH groups in PVA molecular chains can form covalent Ti–O–C bonds,^11^ which keeps the PVA matrix stable in solution. While some of the characteristic peaks of TiO_2_, PVA and PEDOT:PSS overlap in FTIR spectrum, the peaks at 955 cm^−1^ and 915 cm^−1^ can be assigned to the C–S bond of the thiophene ring in PEDOT (the S=O and O–S–O signals of the PSS chains are hidden by strong PVA polymer absorption).^42^ The peaks at 2910 cm^−1^ and 3230 cm^−1^ indicate C–H stretching and O–H functional groups in PVA.^43^ These peaks are broader in P samples when compared to those appearing in T and PT, pointing to the fact that heat treated T and PT films are more crystalline in nature. It also supports the occurrence of Ti–O–C bonds in T and PT films.

Raman spectroscopy was performed to improve the understanding and to provide structural information of the P, T and PT films. Fig. 4(b) reveals the vibrational modes of the films. In the Raman spectrum of the P sample, the vibrational mode at 990 cm^−1^ was assigned to the deformation of oxyethylene rings, while the vibrational modes at 1100 cm^−1^ and 1218 cm^−1^ were attributed to the PSS component and PEDOT C_α_–C_α′_ inter ring stretching vibrations, respectively.^44^ The peaks around 1369 cm^−1^ and 1434 cm^−1^ were assigned to the C_β_–C_β_ stretching and C_α_=C_β_ asymmetrical vibration of PEDOT, respectively. The peaks at 1508 cm^−1^ and 1560 cm^−1^ correspond to the C_α_=C_β_ vibration of the PEDOT and PSS component.^44^ Four characteristic active Raman modes of TiO_2_ with symmetries *E*_g_, *B*_1g_, *A*_1g_ and *E*_g_ were observed in the T film at about 144 cm^−1^, 399 cm^−1^, 520 cm^−1^, and 638 cm^−1^, respectively.^45^ All the PEDOT:PSS and TiO_2_ characteristic peaks were observed in the PT-film, which implies successful formation of a TiO_2_–PEDOT:PSS hybrid heterojunction in a PVA matrix.

### 3.1 Photocatalytic activity of the PVA/TiO_2_ and PVA/PEDOT:PSS + TiO_2_ hybrid films

**Fig. 5:**
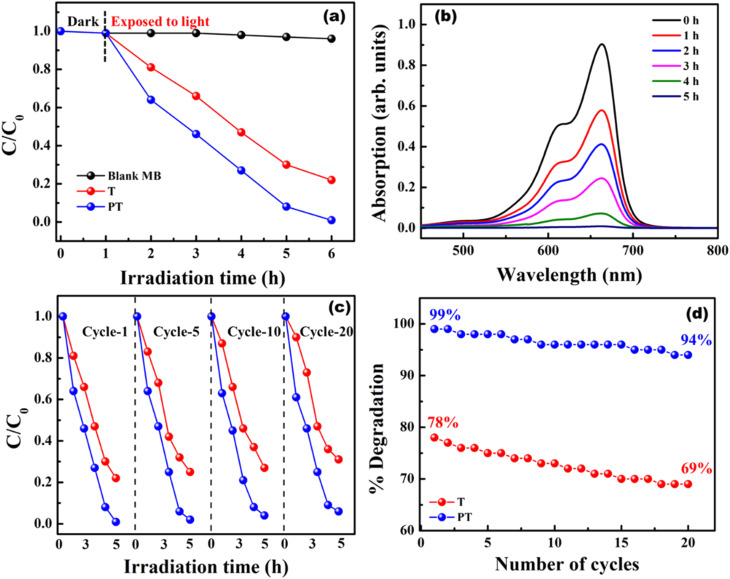
(a) Concentration (*C*/*C*_0_) profile of MB using T and PT films for degradation, (b) degradation profile of MB using a PT film, (c) reusability of the T and PT films for degradation of MB for twenty cycles, and (d) degradation of MB with cycling.

**Fig. 6:**
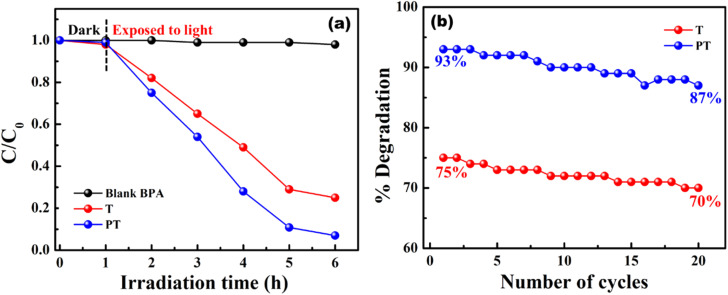
(a) Concentration (*C*/*C*_0_) profile of BPA using T and PT films for degradation, and (b) degradation of BPA with cycling.

Model wastewater containing organic effluents like MB and BPA could be degraded using PVA/TiO_2_ and PVA/TiO_2_–PEDOT:PSS hybrid films under simulated sunlight. The photocatalytic performance of a PVA/TiO_2_–PEDOT:PSS hybrid heterojunction film was analyzed and compared with a PVA/TiO_2_ film. Initially 10 ppm of MB dye solution was prepared, and 1.5 cm × 1.5 cm films were dipped in 20 ml of effluent solution and kept in the dark for 1 h to achieve an adsorption–desorption equilibrium. Film T showed a photodegradation efficiency of about 78%, whereas film PT exhibited about 99% photodegradation under light irradiation (1000 W m^−2^, AM1.5) for 5 h. The concentration and degradation profile of MB using T and PT are shown in Fig. 5(a and b). The enhanced photocatalytic performance of the PT film is due to the formation of a heterojunction between the wideband gap TiO_2_ (∼3.2 eV) and the lower bandgap PEDOT:PSS (∼1.6 eV). Formation of a heterojunction enables the absorption of a broader-spectrum light as well as effective charge separation, thus mitigating the electron–hole pair recombination. Immobilization of TiO_2_–PEDOT:PSS through use of a PVA matrix enables recycling for multiple treatment cycles without any need of powder extraction. The as prepared films are highly stable over 20 cycles and the photodegradation efficiency is consistent in all cycles for over 100 h. The degradation performance of the T and PT films for multiple cycles is shown in Fig. 5(c and d). After 20 cycles the degradation efficiency drops to 69% and 94% for the T and PT films, respectively. PVA matrix retained TiO_2_ and PEDOT:PSS for several cycles, hence the films are extremely stable, which is very important for efficient photocatalytic activity for multiple cycles. Another organic effluent, bisphenol-A (10 ppm), was also degraded using T and PT films. The degradation performance of the films along with their respective cyclic stability is shown in Fig. 6(a and b). The degradation efficiency of T and PT are 75% and 93%, respectively. After 20 cycles the degradation efficiency has dropped to 70% and 87% for the T and PT films, respectively. The films showed almost consistent performance in all the cycles for degrading BPA.

**Fig. 7:**
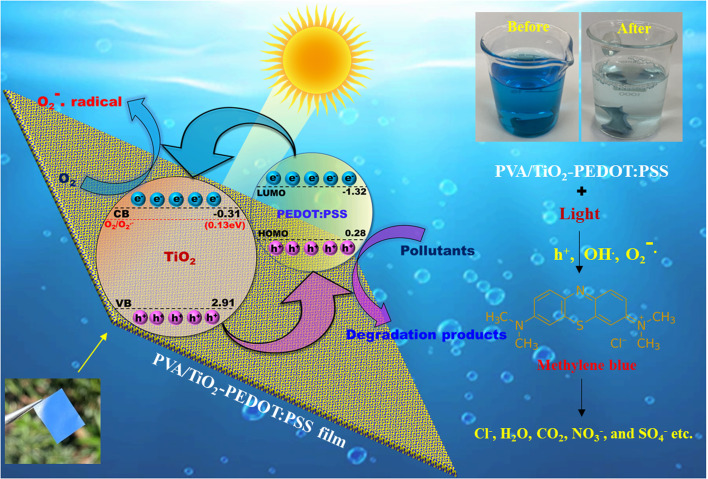
A plausible mechanism of photodegradation of organic effluent using a TiO_2_–PEDOT:PSS heterojunction catalyst.

A plausible mechanism to explain the degradation is shown in Fig. 7. In principle, photocatalytic degradation occurs when a TiO_2_–PEDOT:PSS heterojunction catalyst is irradiated with light and produces electron–hole (e^−^–h^+^) pairs, which react with H_2_O, OH^−^ and O_2_ to generate oxidizing species like hydroxyl radicals (˙OH), superoxide radicals (O_2_˙^−^) and hydrogen peroxide (H_2_O_2_), *etc*.^46^ According to previous reports, the positions of the highest occupied molecular orbital (HOMO) level and lowest unoccupied molecular orbital (LUMO) level of PEDOT:PSS are located at 0.28 eV and −1.32 eV, respectively,^47^ while the estimated positions of the valence band (VB) and conduction band (CB) of TiO_2_ are approximately 2.91 eV and −0.31 eV, respectively.^48^ The corresponding band alignments of the two components are illustrated in Fig. 7. Due to the well-matched energy levels, photo-generated holes of TiO_2_ can be easily transferred to the HOMO level of PEDOT:PSS. Being an excellent hole conductor, PEDOT:PSS can transport the holes to the surface of the photocatalyst quickly, which hinders their recombination with electrons and participate in the oxidation of dye molecules. On the other hand, the electrons of PEDOT:PSS can immigrate to the conduction band of TiO_2_. Since the conduction band edge of TiO_2_ (−0.31 eV) is more negative than the reduction potential of O_2_/O_2_˙^−^ (0.13 eV), the electrons from the conduction band can react with oxygen molecules and produce superoxide radicals (O_2_˙^−^) which subsequently take part in the dye degradation. These redox reactions decompose the organic effluents into harmless byproducts such as CO_2_, H_2_O, *etc.* Excessive OH^−^ groups on the PVA surface also serve as active species for efficient degradation of the organic effluents.

## 4. Conclusion

PVA/TiO_2_–PEDOT:PSS films were successfully fabricated by a simple and convenient method combining solution casting followed by heat treatment. The photocatalyst showed good stability due to strong interaction and Ti–O–C covalent bond formation between the PVA matrix and TiO_2_, originating from the heat treatment. The addition of PEDOT:PSS establishes a hybrid heterojunction with TiO_2_ (PVA/TiO_2_–PEDOT:PSS), enhancing the light absorption as well as charge separation, which improves the photocatalytic performance over PVA/TiO_2_. The photodegradation efficiency of these films remains stable during twenty subsequent cycles, for over 100 h, which puts forth the immobilized PVA/TiO_2_–PEDOT:PSS hybrid heterojunction photocatalysts as promising candidates for industrial wastewater treatment.

## Conflicts of interest

There are no conflicts to declare.

## Supplementary Material

RA-013-D2RA06729C-s001
