# Supplementary material for: Immobilization of a TiO2–PEDOT:PSS hybrid heterojunction photocatalyst for degradation of organic effluents
Source: RSC Adv. 2023 Jan 19;13(5):3095–101. doi: 10.1039/d2ra06729c (PMC9850944; doi:10.1039/d2ra06729c)
Supplement: RA-013-D2RA06729C-s001 [file RA-013-D2RA06729C-s001.pdf]

Electronic Supplementary Material (ESI) for RSC Advances.
This journal is © The Royal Society of Chemistry 2023

Supporting information

Immobilization of TiO_2-PEDOT: PSS hybrid heterojunction photocatalyst for degradation of organic effluents

Durga Sankar Vavilapalli[1,2,], Johanna Rosen[1], Shubra Singh[2]*

[1]Materials Design, Department of Physics, Chemistry and Biology (IFM), Linköping University, SE-581 83 Linköping, Sweden.

[2]Crystal Growth Centre, Anna University, Chennai-600025, India.

**Corresponding author email: durga.sankar.vavilapalli@liu.se*

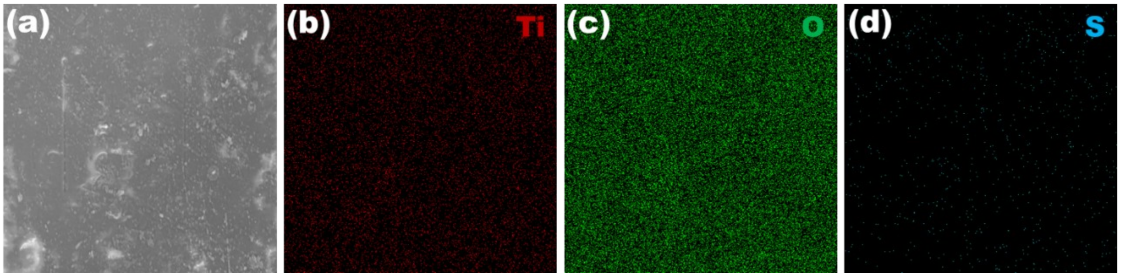

Figure S1: EDS mapping of a TiO_2-PEDOT:PSS (PT) film showing a uniform distribution of Ti, O and S elements.

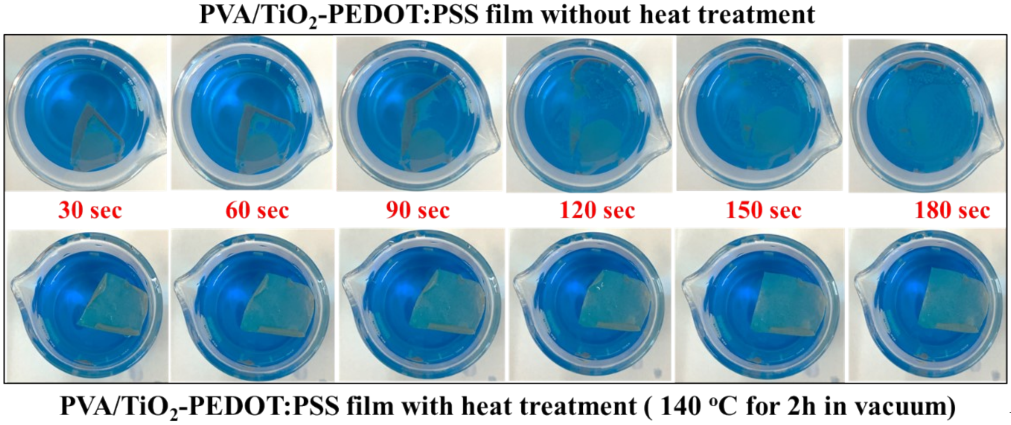

Figure S2: Images showing the stability of the film (with and without heat treatment) at various time intervals. The film without heat treatment disintegrated within three minutes after being in contact with an aqueous dye solution, while the heat-treated film was stable for more than 100 hrs.

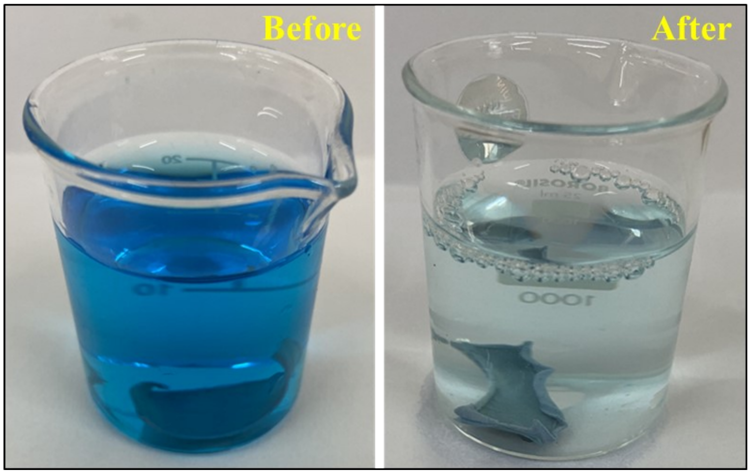

Figure S3: Stability of a PVA/TiO_2-PEDOT:PSS membrane after 20 cycles (> 100 hrs).

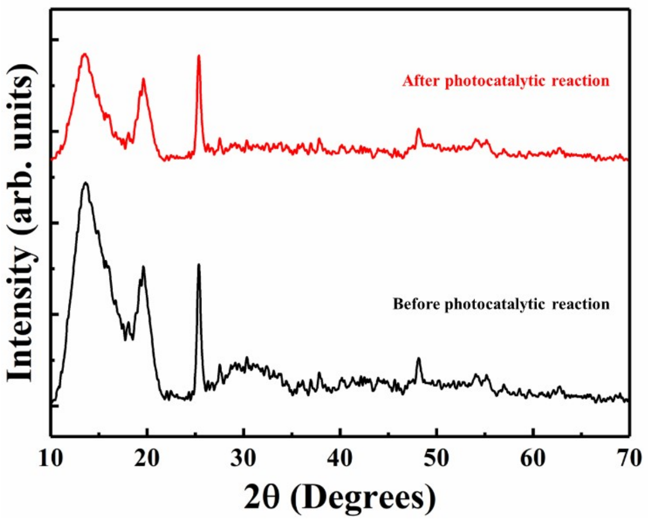

Figure S4: XRD patterns of PT-film, before and after the 20 cycles of photocatalytic degradation of MB.
